# Mortality and Pathology Associated with Highly Pathogenic Avian Influenza H5N1 Outbreaks in Commercial Poultry Production Systems in Nigeria

**DOI:** 10.1155/2014/415418

**Published:** 2014-10-22

**Authors:** Olatunde Babatunde Akanbi, Victor Olusegun Taiwo

**Affiliations:** ^1^Central Diagnostic Laboratory, National Veterinary Research Institute, PMB 1, Vom 930001, Plateau State, Nigeria; ^2^Department of Veterinary Pathology, Faculty of Veterinary Medicine, University of Ibadan, Ibadan 200284, Oyo State, Nigeria

## Abstract

Commercial layer-type, pullet, cockerel, and broiler chicken flocks infected with highly pathogenic avian influenza (HPAI) H5N1 in Nigeria between 2006 and 2008 were investigated for morbidity, mortality, and pathology. Of the one hundred and fifty-three (153) farms confirmed with HPAI infection, one hundred and twenty-seven (127) were layer-type farms, nine (9) were pullet and broiler farms each, and eight (8) were cockerel rearing farms. This study revealed the morbidity and mortality of a total of 939,620 commercial layer chickens, 16,421 pullets, 3,109 cockerels, and 6,433 broilers. Mortality rates were 11.11% in commercial layers, 26.84% in pullets, 45.51% in cockerels, and 73.92% in broilers in a total of eighteen (18) states and the Federal Capital Territory, Abuja. A total of 316 carcasses were examined of which 248 were commercial layer, 25 were pullet, 14 were cockerel, and 29 were broiler. Main clinical and pathologic findings were observed in the nervous, circulatory, respiratory, integumentary, musculoskeletal, hemopoietic, gastrointestinal, and reproductive systems and, occasionally, lesions were generally nonspecific and multisystemic. Lesions occurred more frequently, severely, and in most of the carcasses examined, irrespective of chicken type.

## 1. Introduction

The index HPAI-H5N1 was confirmed in Nigeria at a commercial poultry farm in Kaduna state, and, by the end of the initial outbreak, over 46,000 poultry [1, 2] had been destroyed. This outbreak brought the Asian strain of highly pathogenic avian influenza (HPAI) H5N1 into Africa for the first time in the beginning of January, 2006 [3–7] in Nigeria. Nigeria lost 945,862 birds of various species at mid-January [8], 2007. Since its emergence in Africa in 2006, avian influenza viruses of the H5N1 subtype have spread rapidly to poultry farms in several African countries. It has caused deaths of millions of birds in Africa [9], as is the case in other affected parts of the world. Highly pathogenic avian influenza (HPAI) is caused by AIVs that are extremely virulent [10], causing up to 100% mortality in domestic chickens. The H5N1 highly pathogenic avian influenza (HPAI) virus has been a great concern not only for the poultry industry but also for human health [11] since 1997. Following the index HPAI-H5N1 in Nigeria, several efforts have been directed to increased surveillance and diagnosis of the disease. This has led to increased diagnostic capacity, reporting, and research into the diverse genotype of H5N1 isolates from Nigeria. The commercial poultry production system in Nigeria as categorized by the FAO [12], which stems from the two major systems: rural poultry production and commercial poultry production, was affected by the HPAI outbreaks. Also, several attempts were made to determine the losses in terms of the number of poultry, monetary, and social values as a result of the disease burden [8] in Nigeria. These attempts have been at some point in the course of the disease [8] or aimed at a particular region of the country [13] due to some identified constraints. Also no overall, detailed clinical and pathological record has been given on a case by case basis for all the outbreaks as seen from different states and regions of the countries which could highlight factors involved in the spread of the disease. In early 2006, Joannis et al. 2006 [4], in their letter to the Veterinary Record, mentioned a few lesions seen in the index case. In addition, Adene et al. 2006 [2] described the clinicopathological and husbandry features associated with the maiden diagnosis of AI in Nigeria. Other reports were limited to cases seen in 2006 [14, 15] only. Akanbi et al. 2007 [5] had earlier reported lesions which depict circulatory disturbances, respiratory, intestinal, and reproductive system pathology in selected cases. This study aimed at achieving the determination of the losses on a state by state basis and the total losses recorded during the entire course of the disease in Nigeria and also aimed at highlighting the morbidity, mortality, and pathology in the commercial poultry production systems as practiced in Nigeria.

## 2. Materials and Methods

*Data.* All the data including state, location, farm flock size, and morbidity and mortality records used in this study were supplied directly by clients who reported and submitted carcasses of layers, pullet, cockerels, and broilers for postmortem examination and avian influenza diagnosis at the National Veterinary Research Institute (NVRI), Central Diagnostic Laboratory, Vom.

*Carcasses.* A total of three hundred and sixteen (316) carcasses from one hundred and fifty-three (153) farms from eighteen (18) states and the Federal Capital Territory, Abuja, were examined. These carcasses were submitted directly by clients for postmortem examination and avian influenza diagnosis and were selected based on laboratory confirmation of HPAI H5N1 virus infection at the NVRI laboratory.

*Pathology.* Carcasses of the commercial birds that died after natural infection with HPAI were submitted for pathological examination. Following postmortem examination of the carcasses, sections of liver, heart, spleen, kidney, lung, trachea, proventriculus, gizzard, duodenum, ileum, cecum, and brain were removed and fixed in 10% buffered formalin. All tissue samples were then embedded in paraffin, sectioned at 5 *μ*m, mounted on clean glass slides, and stained with hematoxylin and eosin (H&E) stains for histopathologic examination using low and high powered field of Carl Zeiss or Nikon binocular microscope.

*Virus Isolation.* For virus isolation, samples of liver, heart, spleen, kidney, lung, trachea, duodenum, and caecum were removed and submitted for virological investigation, where the homogenates of the trachea, lung, liver, spleen, and kidney were inoculated via the allantoic cavity into 9–11-day-old embryonating chicken eggs [16] obtained from specific antibody negative chickens.

## 3. Results

### 3.1. Data Set Distribution

During the period under review (2006–2008), a total of eighteen (18) states (Adamawa, Bauchi, Bornu, Jigawa, Kaduna, Kano, Katsina, Sokoto, Plateau, Nasarawa, Kwara, Lagos, Ogun, Oyo, Rivers, Edo, Anambra, and Enugu) and the Federal Capital Territory, Abuja, had confirmed HPAI H5N1 outbreaks in commercial layer, pullet, cockerel, and broiler (Table 1). A total of one hundred and fourteen thousand, nine hundred and twenty-nine (114,929) commercial chickens died from a total flock size of nine hundred and sixty-five thousand, five hundred and eighty-three (965,583) before the rest of the flocks were stamped out. The north-western region recorded the highest number of mortalities in commercially raised chickens as a result of natural infection with HPAI in Nigeria. The figure (69,741) represents 60.68% of the total number (114,929) of commercially raised chickens that died as a result of HPAI (Table 1). While the total number of chicken losses as a result of HPAI death and stamping out in this region (438,386) was second to the south-western region where commercial bird losses totaled 445,320 and the number that died as a result of HPAI when the reporting was done was 26,878, being 23.38% of the total number of commercial chickens that died as a result of HPAI.

### 3.2. Mortality

Mortality rates were the highest in broilers flocks (73.92%) and the least in layers flocks (11.11%). The mortality rate was 45.51% in cockerels and was 26.84% in pullet. A breakdown of the figures, which showed layer flocks, has been the most affected commercial chicken type with a total bird loss of 939,620, representing 97.31% of the commercially raised chickens. Of the 939,620 layer population affected in 127 farms, 104,351 layers, representing 90.79% of 114,929, died as a result of direct HPAI infection in layer flocks. Eight hundred and fifty thousand, six hundred and fifty-four (850,654) birds, which represent the difference between the flock size and the number of the dead, were stamped out. Broilers were the second most affected commercial chicken type of which 6,433, representing 0.66% of the total flock size (965,583) of commercially raised chickens, were affected by HPAI. Of the 6,433 broilers affected, 4,755 broilers, representing 4.14% of the total number of commercially raised chickens (114,929), died as a result of direct HPAI infection, while the remaining broilers were stamped out. Pullets were the next affected commercial chicken type of which 16,421 (11.70%) were affected and 4408 (3.84%) were reported to be dead. Cockerel was the least affected commercial chicken type of which 3109 (0.32%) were affected and 1,415 (1.23%) were reported to be dead.

### 3.3. Clinical Signs and Pathology

**Figure 1:**
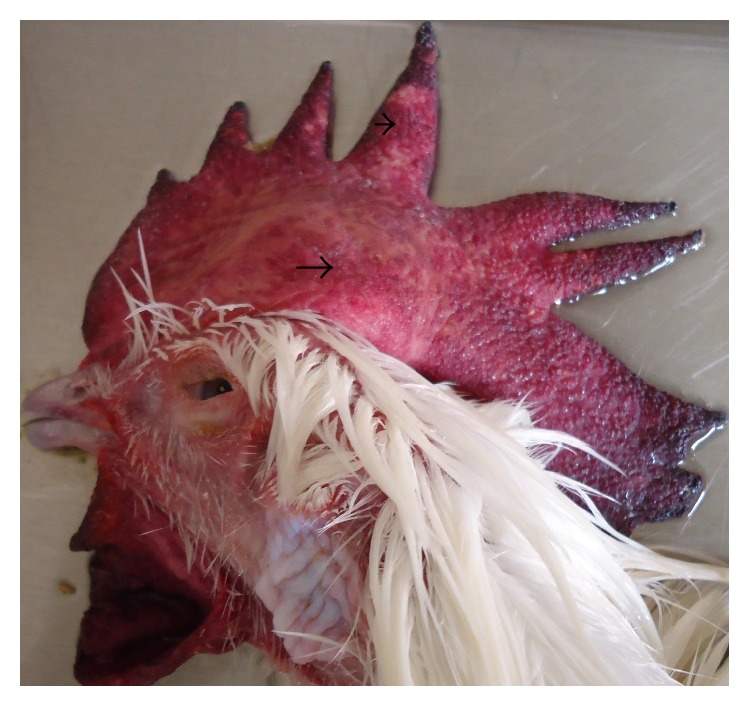
Layer chicken, head showing swollen comb (arrow) and congestion (arrow head).

**Figure 2:**
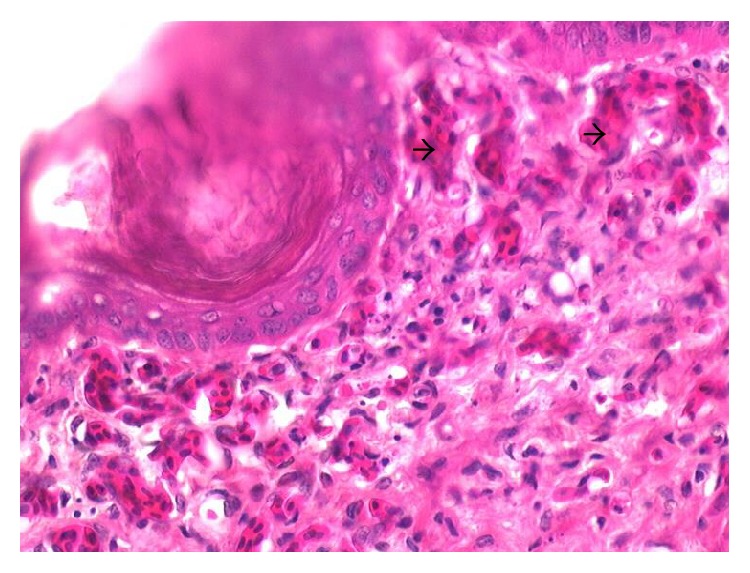
Layer chicken, comb showing severe dermal vascular congestion (arrow heads) H&E, ×400.

**Figure 3:**
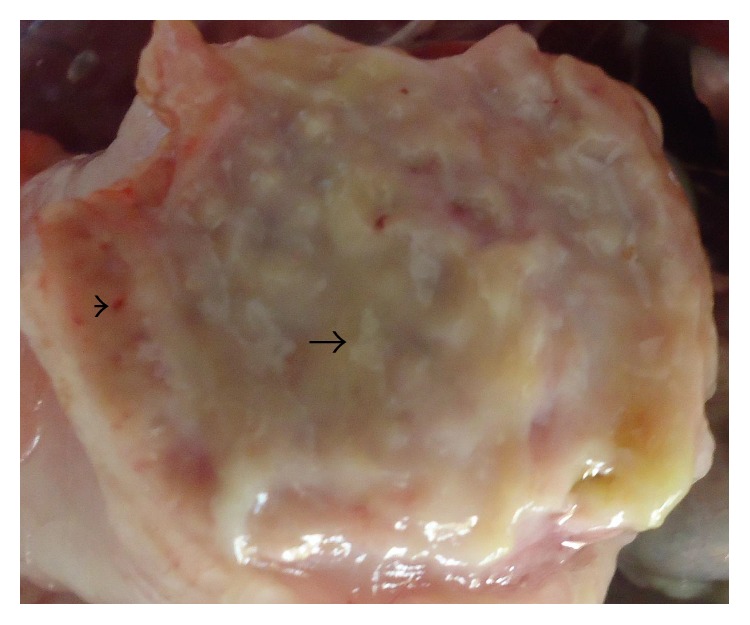
Layer chicken, proventriculus showing swollen glands with excess mucous exudates (arrow) and mild multifocal to diffuse petechiae hemorrhages (arrow head).

**Figure 4:**
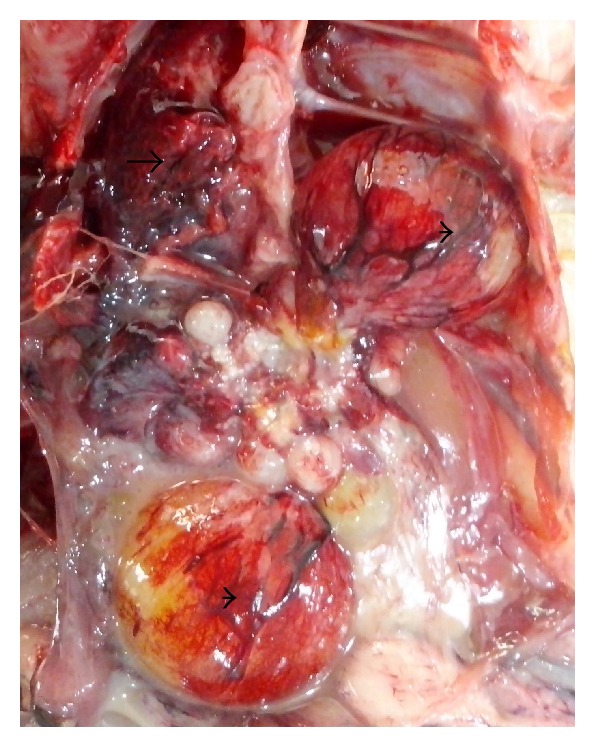
Layer chicken, thoracic and abdominal cage, showing congested and consolidated lungs (arrow) and haemorrhagic ovarian follicles (arrow head).

Main clinical and pathologic findings were observed in the nervous, circulatory, respiratory, integumentary, musculoskeletal, gastrointestinal, and reproductive systems and occasionally lesions are multisystemic. Signs observed and reported are sudden death, high mortality, weakness, and recumbency. Others ranged from nasal discharges, dyspnea, coughing, sneezing, diarrhea, shank hyperemia and haemorrhage, inability to stand, ataxia, and torticollis. In layers, egg structural abnormalities such as shell-less egg, white-colored eggs, and soft eggs were reported. Lesions observed in the circulatory system included congestion (Figures 1 and 2) and cyanosis of comb and wattle, comb and wattle edema, and facial and subcutaneous edema. Within the respiratory system, there were airsacculitis and pneumonia. There was petechiation to ecchymoses of the proventricular (Figure 3) and intestinal mucosa with resultant enteritis in the gastrointestinal system. Integumentary system lesions are mainly cyanosis, edema, and ecchymotic hemorrhages while there were inflammatory, degenerative, and necrotic lesions in the musculoskeletal system. In adult birds, mainly layers reproductive lesions were observed and they were mainly ovarian follicular ecchymotic hemorrhages (Figure 4).

**Figure 5:**
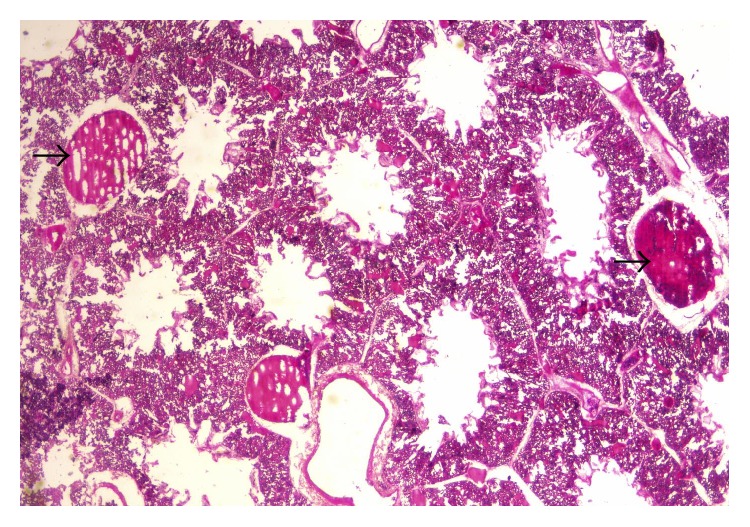
Layer chicken, lung showing severe multifocal vascular congestion (arrow), H&E, ×400.

**Figure 6:**
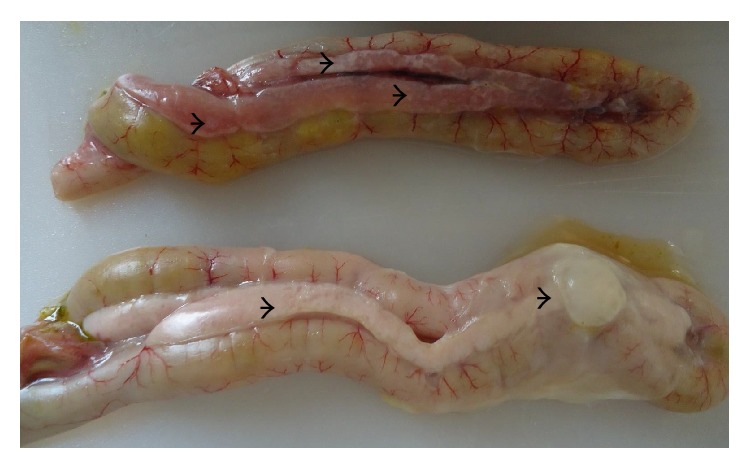
Layer chicken, duodenal loop showing multifocal necrosis on the pancreas (arrow heads).

**Figure 7:**
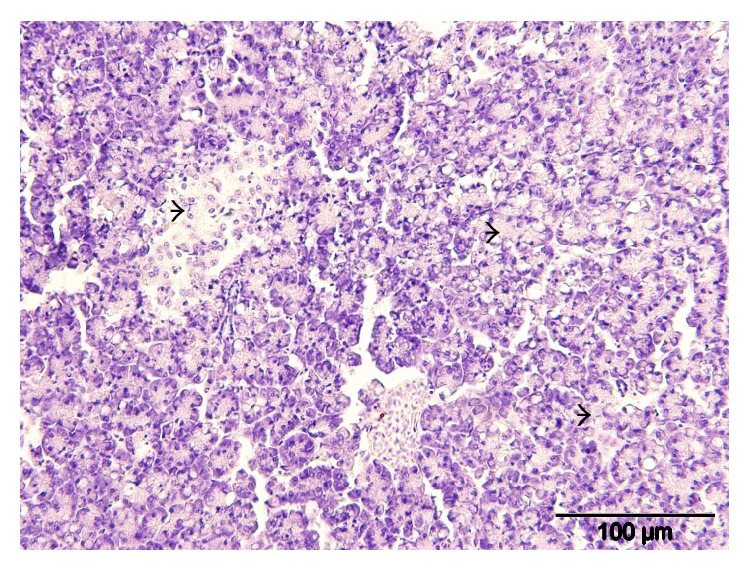
Layer chicken, pancreas showing diffuse necrosis of acinar cells of the pancreas (arrow heads) H&E.

**Figure 8:**
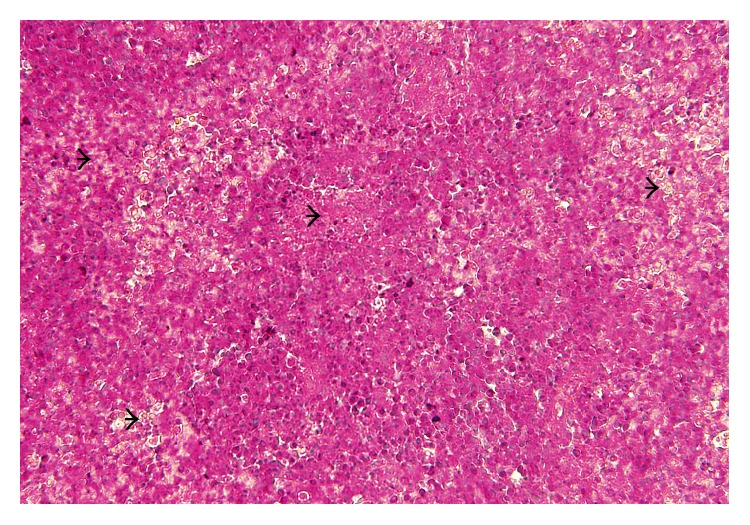
Layer chicken, spleen showing severe diffuse lymphoid necrosis and depletion (arrow heads) H&E, ×400.

**Figure 9:**
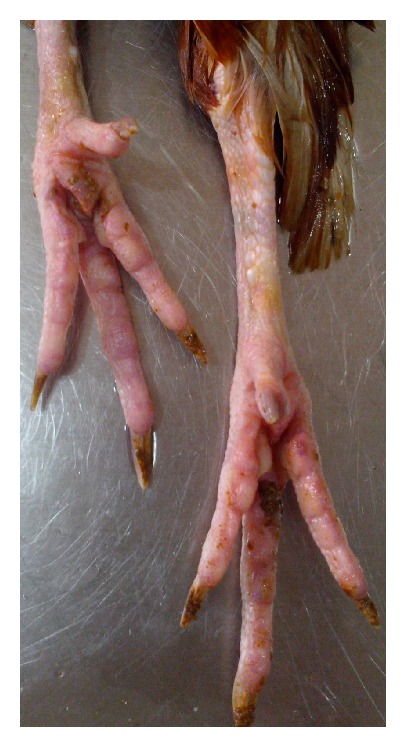
Layer chicken, shank showing hyperemia.

*Layers.* Most of the commercially raised layers (67%) exhibited lesions of the circulatory system, mainly cyanosis of comb and wattle with occasional facial edema. Only 20% showed nervous signs and brain lesions of neuronal and Purkinje cell necrosis of cerebrum and cerebellum, respectively. Respiratory lesion of airsacculitis and pneumonia (Figure 4) with vascular congestion (Figure 5) was evident in 43.5% with more than half having pneumonia. Of the 70 clinical reports of diarrhea, only 42 had enteritis and 18 were hemorrhagic. Enteric petechiation and ecchymoses were observed in 45 of the carcasses while pancreatic necrosis was occasionally encountered (Figures 6 and 7). Splenic necrosis and loss of lymphoid cells were also seen in the carcasses (Figure 8). A few carcasses (6.4%) showed integumentary system lesions; these are mainly cyanosis, edema, and ecchymotic hemorrhages. Only 13.7% had muscular hemorrhages with necrosis, and/or myositis. Reproductive lesions were only observed in these layers, and these were observed in 15.3% only. These were mainly ovarian follicular ecchymotic hemorrhages and structure abnormality. Forty-one (41) percent of the layers had multisystemic lesions. Shank hyperemia (Figure 9) and haemorrhage were also frequently seen.

*Pullet.* Of the twenty-five (25) pullet carcasses examined, 60% had report of the circulatory system signs, mainly cyanosis of comb and wattle with occasional facial edema. Only 4% showed nervous lesion in the brain such as neuronal and Purkinje cell necrosis of cerebrum and cerebellum, respectively. Respiratory lesions of nasal exudation, airsacculitis, and pneumonia were evident in 32%. Enteric lesion was observed in 3 (12%) of the 25 carcasses examined. Twenty-eight (28) percent had muscular and shank hemorrhages with necrosis, and/or myositis. Twenty-four (24) percent of the pullets which died suddenly were from flocks with high mortality and also had multisystemic lesions. No lesion was observed in the integumentary and reproductive systems, respectively, in all carcasses examined.

*Broiler.* Of the twenty-nine (29) broiler carcasses examined, 82.7% had lesions of the circulatory system, mainly cyanosis of comb and wattle with occasional facial edema. Only 4 (13.7%) showed brain lesions of neuronal and Purkinje cell necrosis of cerebrum and cerebellum, respectively. Respiratory lesions of airsacculitis and pneumonia were evident in fourteen (48.2%) carcasses. Enteric lesion was observed in 9 (31%) of the 29 carcasses examined. Five (17.2%) carcasses had muscular and hemorrhages with necrosis and/or myositis. Ten (34.4%) of the broilers which died suddenly were from flocks with high mortality and had multisystemic lesions. No lesion was observed in the integumentary and reproductive systems, respectively, in all carcasses examined.

*Cockerel.* Of the fourteen (14) cockerel carcasses examined, 64% had lesions of the circulatory system, mainly cyanosis of comb and wattle with occasional facial edema. Only 5 (35.7%) showed nervous lesions of neuronal and Purkinje cell necrosis of cerebrum and cerebellum, respectively. Respiratory lesions of airsacculitis and pneumonia were evident in five (35.7%) carcasses. Enteric lesion was observed in 6 (42.8%) of the 14 carcasses examined. Eight (57.1%) carcasses had muscular and shank hemorrhages with necrosis and/or myositis. Five (35.7%) of the pullets which died suddenly were from flocks with high mortality and had multisystemic lesions. No lesion was observed in the integumentary and reproductive systems in all carcasses examined.

## 4. Discussion

The north-western region recorded the highest number of mortalities in commercially raised chickens as a result of natural infection with HPAI in Nigeria. This represents 60.68% (69,741) of the total number of chickens which died as a direct result of HPAI infection. The total number of chickens which died as a direct result of HPAI infection and those that were stamped-out in this region was 438,386, only second to the total number of chickens which died as a direct result of HPAI infection and those that were stamped out in the south-western region which stood at 445,320. Indeed, south-western Nigeria, particularly the states surrounding the city of Lagos [17], holds much of Nigeria's poultry industry. It is estimated that over 65% of Nigeria's commercial poultry are located in the five southern states [17] of Lagos, Ogun, Oyo, Osun, and Ondo. A very high number of poultry deaths and losses were also reported in a north-western regional [13] avian influenza investigation in the north-western region, apart from the fact that it was in this region where the first outbreak of HPAI in Nigeria was reported [4, 18] in a farm that had predominantly commercial birds [2, 14]. Although the total poultry losses were higher in the southwest region, the total number of chickens which died as a direct result of HPAI infection was 26,878 being 23.38% of the national total number of commercially raised chickens that died as a direct result of HPAI infection, as compared with 60.68% (69,741) in the northwest. This difference may be as a result of the fact that the farms in the southwest are more organized and densely stocked as compared with those in the northwest which are not well organized and not as densely stocked. The finding of high mortality in the north-western states is consistent with earlier reports [2, 13]. In the north-western regional analysis [13] which excluded bird losses in Sokoto state, four hundred and eighty thousand, three hundred and seventy-eight (480,378) birds were reported as being lost as a result of HPAI incursions and stamping out. The deviation of this finding from ours may be as a result of nonseparation between the various sectors of poultry production systems, as it includes backyard flocks and noncommercial and commercial flocks. The north-central states had a mortality of 6418 (5.58%) being the total number of commercial chickens that died as a result of HPAI when the reporting was done, while the total number of chicken losses as a result of natural death and stamping out policy in this region was 31,189. The south-south region lost more birds to direct HPAI infection (5309) as compared to the north-eastern region (4880) but more birds (29586) were destroyed in the northeast than in the south-south where 7,700 birds were destroyed. The southeast lost the least number of birds to HPAI (1703) but had a high number of birds' losses to HPAI and stamping out (13402). Under the commercial poultry production sector practiced in Nigeria [17], the commercial layer-type chicken was the most hit by the highly pathogenic avian influenza outbreaks experienced by the Nigerian poultry industry. With a total flock size of 939,620 lost in 127 farms having confirmed outbreaks, the commercial layer-type chicken suffered a major economic loss and devastation. This is attributed to the large number of laying birds in individual flocks as compared to other chicken types under these sectors. This significantly affected the economy not only because a whooping sum of N631 million (US$5.43 million) was paid as compensations to farmers [19] but also because laying hens contribute huge resources to the national poultry flock and this emphasizes the importance of commercial layer flocks [20] for Nigeria's economy. It was also observed that the average mortality rate was the least in commercial layers (11.11%) and the highest in broilers (73.92). This may be as a result of the cage housing of commercial layers which restrict movement and contact between HPAI infected ones and noninfected ones compared with broilers raised in deep litter and which also share water and feed sources. Average mortality rate was higher in cockerels (males = 45.51%) than in pullets (females = 26.84%). The necropsy finding observed in the 316 carcasses examined of which 248 were commercial layer, 25 were pullet, 14 were cockerel, and 29 were broilers cut across more than one system and occurred more frequently and severely and in most of the carcasses examined irrespective of chicken type. In commercial layers, the systems affected included mainly circulatory, respiratory, and intestinal systems as observed by earlier investigators from the index case of HPAI [2, 4] in Nigeria. Only 20% of the 248 layer carcasses had neurologic signs and lesions contrary to what was observed in the index case where only young stocks had nervous system signs [2]. Also, other investigators reported presence of nervous signs and lesion with no particular reference to age [4] and with reference to adult birds [15]. Eighty-two percent (82.7%) of the broilers showed signs and lesions of circulatory disturbances followed by 67% in layers. More broilers and cockerels showed neurological signs and lesion more than in layers. This work was able to document the total HPAI bird losses in commercially raised chickens in Nigeria on a state by state cases and region by region cases, previously unreported. It also showed that mortality was the highest in broiler and males (cockerels) had more death than females (pullets).

## 5. Conclusion

This study was able to document the losses in HPAI infected commercial chickens in Nigeria and the overall pathological findings of HPAI infection in chickens in Nigeria previously unreported.

## Figures and Tables

**Table 1:** Commercial poultry losses in HPAI infection in Nigerian states and region.

*S*/*N*	Zone	State	Flock size	Number of the dead	Chicken type
	Northeast	Adamawa	55	36	Cockerel
		Bauchi	28731	4637	Layer, pullet, broiler
		Bornu	800	207	Layer, pullet, cockerel
	Regional subtotal		**29586**	**4880**	

	Northwest	Jigawa	13786	3000	Layer
		Kaduna	87435	51333	Layer, pullet, broiler, cockerel
		Kano	234998	7930	Layer, cockerel
		Katsina	95605	6778	Layer
		Sokoto	6562	700	Layer
	Regional subtotal		**438386**	**69741**	

	North central	Abuja	236	165	Layer, broiler
		Plateau	24210	4712	Layer, pullet, broiler, chick
		Nasarawa	58	56	Broiler, cockerel, mixed
		Kwara	6685	1485	Layer
	Regional subtotal		**31189**	**6418**	

	Southwest	Lagos	291780	21632	Layer, pullet, broiler
		Ogun	138536	642	Layer, cockerel
		Oyo	15004	4604	Layer, broiler, cockerel
	Regional subtotal		**445320**	**26878**	

	South-south	Rivers	1200	200	Layer
		Edo	6500	5109	Layer, pullet, broiler
	Regional subtotal		**7700**	**5309**	

	Southeast	Anambra	13352	1653	Layer
		Enugu	50	50	Broiler
	Regional subtotal		**13402**	**1703**	
	National total		**965583**	**114929**	
